# Consistency in self-reported age at first sex and marriage among adolescents and young adults in Northwestern Tanzania: insights from repeated responses

**DOI:** 10.3389/frph.2025.1488604

**Published:** 2025-06-12

**Authors:** Jacqueline Materu, Jim Todd, Emma Slaymaker, Mark Urassa, Milly Marston, Ties Boerma, Eveline T. Konje

**Affiliations:** ^1^ Sexual and Reproductive Health Program, National Institute for Medical Research, Mwanza Centre, Mwanza, Tanzania; ^2^ Department of Biostatistics, Epidemiology and Behavioral Sciences, School of Public Health, Catholic University of Health, and Allied Sciences, Mwanza, Tanzania; ^3^ Department of Population Health, London School of Hygiene and Tropical Medicine, London, United Kingdom; ^4^ Institute for Global Public Health, University of Manitoba, Winnipeg, MB, Canada

**Keywords:** adolescents, sexual risk behaviour, age at first sex, age at first marriage, consistency, variability

## Abstract

**Introduction:**

Adolescents and young adults face unique sexual and reproductive health (SRH) challenges, with early sex and marriage linked to negative outcomes. Reported ages at first sex (AFS) and first marriage (AFM) are crucial indicators for SRH and HIV intervention programs. This study aimed to assess the consistency of AFS and AFM reports among adolescents and young adults with repeated responses across eight survey rounds (1994–2016) from the Magu Health and Demographic Surveillance System (Magu HDSS).

**Methods:**

A serial cross-sectional survey comprising 58,654 observations from 33,177 individuals in the Magu HDSS, conducted between 1994 and 2016, was analysed. Structured face-to-face interviews were used for data collection. A fixed-effects panel regression model was applied to assess within- and between-individual variability. Reported AFS and AFM were categorized as consistent or inconsistent across survey rounds. Variability and consistency were further analysed across different age groups, sexes, residence area, education, pregnancy and HIV status.

**Results:**

The study revealed significant within-individual variability, with nearly half of the variation due to individual-specific reporting changes over time. Among 2,637 individuals aged 15–24 who reported AFS more than once, 1,312 (49.8%) provided consistent values. For AFM, 621 out of 920 individuals (67.5%) reported same age values across multiple surveys. In other words, 49.8% of individuals provided the same AFS values each time, while 67.5% reported the same AFM values; the rest reported different values. Sub-analysis showed that age, sex, residence, HIV status, pregnancy, and education influenced variability and consistency. Females exhibited higher consistency in AFS (56.7%) and AFM (61.0%) compared to males (43.5% and 44.9%, respectively). Adolescents (15–19 years) reported more consistently with lower variability than young adults (20–24 years) and adults (25–49 years).

**Conclusion:**

This study assesses the extent of consistency in reported ages among young individuals and identifies the challenge of self-reported AFS and AFM data due to inherent variability and inconsistency. It highlights the need to scrutinize the consistency of these reported events each time these indicators are used to evaluate trends and progress in SRH and HIV programs. A systematic analytical approach is essential for improving data quality and obtaining accurate estimates.

## Introduction

Self-reported data from surveys on adolescent and young adult sexual behaviour is frequently utilised to shape research and policy agendas on reproductive health issues (1, 2). Accurately measuring the timing of first sexual intercourse (age at first sex, AFS) and the timing of marriage (age at first marriage, AFM) is essential for understanding sexual and reproductive health (SRH) outcomes among young people. These events serve as key markers of sexual maturity and social transition, carrying significant implications for public health (3–5). The age at which adolescent and young adult first engage in sexual activity and when they marry significantly influences their vulnerability to sexually transmitted infections (STIs), unintended pregnancy, and various social and health consequences (3–7). Early marriage has demographic and health ramifications for both individuals and societies (6, 7). Early sexual debut is associated with multiple health risks, including increased sexual activity without condom use raising the risk of HIV and other STIs (8–10), and for women, it exposes them to early pregnancy and childbirth complications (11, 12).

Understanding trends in sexual behavior is essential for evaluating the effectiveness of prevention programmes targeting early sexual debut and marriage (13), preventing sexually transmitted infections (14, 15), and assessing the impact of increased educational access on reproductive health outcomes (16).However, despite the growing focus on sexual health, significant challenges persist in accurately capturing these behaviours over time, particularly in low- and middle-income countries (LMICs), where socio-cultural norms and economic factors strongly influence sexual behaviour. Existing studies often rely on cross-sectional designs, which fail to capture changes in behaviour within individuals and do not account for the influence of socio-demographic factors and reporting biases (17–19).

In sub-Saharan Africa, where cultural norms significantly shape sexual behaviour and marriage patterns, obtaining accurate data on AFS and AFM is even more critical. Yet, self-reported data—despite being the primary source of information on AFS and AFM—are susceptible to various biases, including response bias, social desirability bias, and recall errors (20–23). These biases are particularly pronounced among young people, who may fear judgment or lack the ability to recall events accurately (20–23). Adolescents, in particular, may be more sensitive to the potential consequences of public disclosure of their sexual and marriage experiences, making them more prone to discrepancies and misreporting (24, 25).

Discrepancies in reporting are influenced by societal and cultural norms, literacy levels, access to information, and recall bias (20–23). Studies have shown that consistency in reporting these events is higher in high-income countries (>80%) (26, 27) compared to LMICs (36.5%–50%) (20, 28). Furthermore, gender differences are evident, with males often reporting older ages than females (29, 30), and older individuals demonstrating lower consistency in their reports (27). Although short-term recall is generally expected to improve consistency, some studies indicate that even with longer recall intervals, consistency can remain high. For instance, a 7-year gap study reported 85.4% consistency (26) compared to 22.2%–50% in studies with 1–2-year gaps (20, 31). Such inconsistencies challenge the validity of self-reported data, potentially leading to misinformed public health policies and ineffective interventions.

Given these challenges, it is essential to understand the factors influencing reporting accuracy, which include individual factors (e.g., age, gender), socio-cultural factors (e.g., stigma, social norms), and methodological factors (e.g., survey design, interview methods, data management, and analysis) (24, 31–33). Inconsistencies in self-reported AFS and AFM are not random but are influenced by these multiple factors. Although, several studies have explored reported AFS and/or AFM using individual samples at one or two time points (31–33), only a limited number have examined consistency across three to five time points (34, 35). Most of these studies have focused on adolescents aged 7–19 years (20, 28, 31) or adults in the general population (27, 35), leaving a critical gap in understanding how these reports vary among diverse socio-demographic groups and change over time.

This study aimed to address these gaps by leveraging data from the Magu Health and Demographic Surveillance System (Magu HDSS), which has tracked sexual behaviour and HIV/AIDS data over two decades. By examining AFS and AFM reports from the same individuals across multiple survey rounds, this study uniquely explores the variability and consistency of these self-reports. This approach not only enhances the accuracy of trend analysis but also provides critical insights for refining public health strategies. Specifically, consistency in reported AFS and AFM was analysed both overall and among subgroups, including adolescents, young adults, and other demographic categories. Understanding these inconsistencies is crucial for designing effective sexual health interventions and ensuring that educational and prevention programmes are tailored to diverse populations.

## Materials and methods

### Data source

We analysed data from eight rounds of serological surveys conducted under Magu HDSS, also known as the Kisesa observational HIV cohort study, between 1994 and 2016. Magu HDSS is an open community cohort located 20 kilometres east of the city of Mwanza in Tanzania. The surveys included all persons aged 15 years and above who were listed in the respective HDSS follow-up rounds, with data collected using a structured questionnaire and face-to-face interviews. These were administered by trained, same-sex interviewers in their mid-20s, recruited from the Sukuma ethnic group to enhance cultural familiarity and rapport with individuals. The questionnaires were administered in Swahili or the local vernacular where necessary, maintaining uniformity across rounds. Interviews were conducted in private, temporary huts to ensure confidentiality, with home visits conducted for individuals who could not initially attend. Interviewers underwent specialized training to ensure consistent data collection practices across all rounds. Data collection methods and environments were standardized across all rounds, with surveys held at central village locations or during home visits for eligible individuals (36, 37). The study ensured robust methodological practices to minimize measurement biases and maximize the reliability of results. The final data collection was in 2016, after which no further data were available for inclusion in our analysis.

Survey round three did not collect data on AFS, and two rounds (survey rounds two and three) did not collect data on AFM. However, in all rounds, we have information reported regarding whether individuals experienced the event or not, so we didn't exclude these rounds from our analysis.

Our study focused on analysing data from all age groups who attended at least one of the surveys conducted between 1994 and 2016, but specifically for the young individuals (aged 15–24 years), with a sub-analysis based on adolescents (aged 15–19 years), young adults (aged 20–24 years), and the adults (aged 25–49 years). To assess consistency and variability, we specifically included individuals who appeared in two or more survey rounds in the analysis. Sampling strategies and survey methods used in Magu HDSS have been described in detail elsewhere (36, 37).

### Ethical approval and consent to participate

This study, approved by the Catholic University Health and Allied Sciences (CUHAS) and the Bugando Medical Centre (BMC) Research Ethics and Review Committee (CREC/585/2022), analysed secondary data from the Magu HDSS. The Magu HDSS had ethical approval from the Lake Zone Institutional Review Board (LZIRB), the Medical Research Coordinating Committee of Tanzania, and the London School of Hygiene and Tropical Medicine. Individuals were provided with informed consent regarding the study's objectives, and explicit written consent was obtained before survey interviews. The informed consent process included an oral presentation, allowing individuals to express agreement or disagreement. For individuals aged 15–17, consent was sought from parents or guardians, with minors also required to assent. Eligible individuals were invited to a central location to respond to the questionnaire in private huts. The survey, conducted in Swahili, covered various topics, ensuring participant confidentiality using unique anonymized identification numbers.

### Measures

The surveys collected data on AFS and AFM by asking two questions: whether the respondent had ever had sexual intercourse or ever married, and if so, at what age (in completed years) they first experienced the event. The wording of the questions regarding AFS and AFM was consistent across all survey rounds. The survey also gathered information on the respondent's age, date of birth, interview date, sex, and other survey parameters (not of interest for the current analysis). In these surveys, no distinction was made between formal marriage and cohabiting (living with) a partner.

### Data management for AFS and AFM

**Figure 1:**
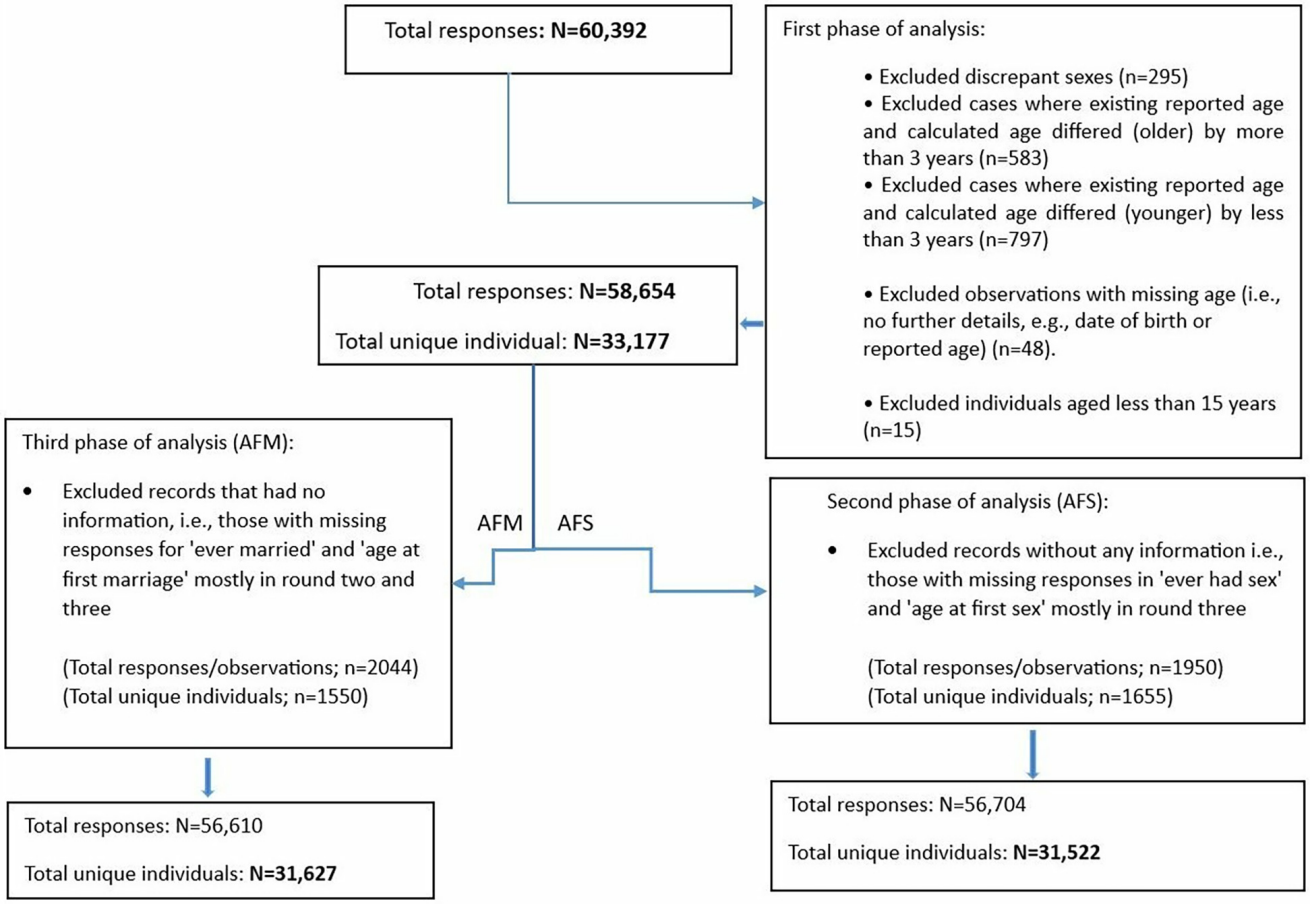
Flow chart of the exclusions in study sample based on age at first sex (AFS) and marriage (AFM).

Data from eight rounds of serological surveys were combined to establish an overall total sample size. During the cleaning process, we removed observations with discrepant sex values across surveys and those with an age less than 15 years, as Magu HDSS considers survey participants to be 15 years and older. We recalculated age based on the interview date and date of birth and compared it with the reported age. Missing age values were replaced with the calculated age, and observations with a discrepancy of more than three years (either younger or older) between the calculated age and reported age were removed, as these may indicate mistaken identity. Additionally, we excluded records with missing age information, where neither a reported nor calculated age was available. After combining data from all eight survey rounds, a total of 60,392 observations from 33,320 records were obtained. Following the cleaning process, the final dataset included 58,654 responses from 33,177 individuals as shown in Figure 1.

### Eligibility and exclusion criteria of the reported AFS and AFM

From the cleaned dataset of 58,654 responses from 33,177 individuals, we applied eligibility and exclusion criteria to define the final data for AFS and AFM analysis. For AFS, individuals with missing information on both ever having had sex and AFS, as well as those who were not asked about these variables, were excluded, resulting in 56,704 observations from 31,522 records. Similarly, for AFM, individuals with missing data on both ever being married and AFM were excluded, resulting in 56,610 observations from 31,627 records (Figure 1).

### Different scenario of reported AFS and AFM and correction methods

Previous studies in Malawi (38), Uganda (39), and other African countries (40–42) have assessed the quality of self-reported sexual behaviour data, including AFS and/AFM, using various methods. These methods include comparing reported values among a sub-sample of re-interviewed survey participants (38), comparing reported values from different surveys among same people with multiple reporters (39), and assessing the consistency against other indicators, such as age at first birth or by comparing behavioural data with biological outcomes like pregnancy (40–42). Other approaches have been used to evaluate and correct inconsistencies in reported AFS, including considering the first reported AFS as correct and disregarding subsequent information, selecting either the higher or lower AFS when respondents report two different values at different times, or taking the average or the most recent reported AFS (31, 35). However, a study population across sequential survey rounds, as in the current study, provides a unique opportunity to assess reporting inconsistencies in AFS and AFM longitudinally and facilitates the development of a systematic approach to address inconsistencies in reported values.

In this study, we identified consistent and inconsistent AFS and AFM data and made corrections whenever necessary. Consistent AFS and AFM data are those that do not change over time. They include data from individuals who have either never experienced events (i.e., sex/marriage) and did not report their AFS or AFM across multiple surveys, or reported AFS and AFM only once across eight surveys, or reported the same AFS and AFM multiple times. Data were also deemed consistent if AFS and AFM were reported multiple times with a difference of, at most, only one year. Inconsistent AFS and AFM data are from individuals who reported AFS and AFM multiple times with variations exceeding one year (i.e., 2, 3, 4, or more years) across the eight surveys. For such cases the mean value was estimated as a proxy for analysis. We further categorized the data into reliable, and unreliable reports, to enhance analytical accuracy. Reliable data include reports that meet one or more of the following conditions: individuals who never experienced the events and consistently did not report AFS and AFM across multiple surveys, those who reported AFS and AFM only once, those who reported AFS and AFM consistently across surveys, or those who reported multiple times but with only one differing value (in such cases, the most common value was considered valid). Reliable data also include cases where AFS and AFM were reported more than once but differed by no more than one year, with the oldest age considered valid. Unreliable data, however, are those deemed insufficiently consistent for use in the analysis, even after attempts to correct for discrepancies. These include reports where AFS and AFM were recorded multiple times with variations exceeding one year (e.g., 2, 3, 4, or more years) and where the mean value was estimated, or cases requiring further investigation to resolve discrepancies (e.g., revisiting original questionnaires or field data). Unreliable data also include instances where individuals reported having experienced the events but did not provide corresponding AFS and AFM values (Supplementary Table S1). We examined the overall consistency and inconsistencies in the reported AFS and AFM, stratifying the data by demographic characteristics. The chi square test was used to determine the significant association between demographic characteristics and consistency rate. A *P*-value of less than 0.05 was considered as statistically significant.

### Variability of the reported AFS and AFM

We utilized a fixed-effects panel regression model (also called within-subject or within-group models) to estimate the variability in reported AFS and AFM. Specifically, we assessed both within-and between-individual variability, as well as the intra-class correlation coefficient (ICC). A *p*-value of less than 0.05 considered statistically significant. This model accounts for clustering within individuals. It is widely used to analyze data that focuses on changes within individuals over time or across groups. By incorporating individual-specific fixed effects, the model controls for unobserved, time-invariant characteristics that may influence responses. This helps ensure a a more accurate estimation of reporting variability (43).

## Results

### Demographic characteristics of the participants

 Table 1 presents the demographic characteristics of the study participants, comprising a total of 58,654 responses from 33,177 unique individuals across eight survey rounds. The number of available responses for certain variables is slightly lower than the total number of participants due to missing data. Overall, the majority of participants were female (57.5%), had completed primary education (65.1%), and resided in rural areas (58.3%). A small proportion of participants had a positive HIV status (5.6%). Nearly half of the participants were younger, with 33.9% aged 15–19 years (adolescents) and 17.0% aged 20–24 years (young adults).

**Table 1:** Demographic characteristics of participants across survey rounds.

Characteristics	Total responses (*N* = 58,654) Individual responses (*N* = 33,177)
Variables	*n* (%)
Sex	
Male	14,104 (42.5)
Female	19,059 (57.5)
Residence area	
Rural	19,351 (58.3)
Semi-urban	13,821 (41.7)
Level of formal education	
No education	7,691 (23.2)
Primary education	21,588 (65.1)
Secondary or higher education	3,865 (11.7)
Age in years	
15–19	11,254 (33.9)
20–24	5,645 (17.0)
25–49	13,006 (39.2)
50+	3,272 (9.9)
HIV status	
Negative	27,510 (82.9)
Positive	1,869 (5.6)

### The variability of the reported AFS and AFM among individuals with multiple reports

 Table 2 shows the variability of reported AFS and AFM among individuals with multiple reports, excluding those who reported only once. Within-individual variability was higher than between-individual variation, with 11.2 for AFS and 12.1 for AFM, compared to 9.9 for AFS and 10.3 for AFM. Approximately 40.0% of the total variance in both AFS and AFM was attributed to individual-specific differences. By age group, adolescents showed lower within-individual variability for AFS (2.0) compared to young adults (3.1). Overall, younger individuals (15–24 years) exhibited lower variability in reporting AFS and AFM compared to older individuals (25–49 years) (Table 3). Variability in AFS and AFM by demographic characteristics and HIV status overall showed higher within-individual variation than between-individual variation across most variables (Supplementary Table S2).

**Table 2:** Overall variability of reported age at first sex (AFS) and marriage (AFM) among multiple reporters.

AFS (responses = 29,991; Individuals = 12,897)		AFM (responses = 21,513; Individuals = 10,112)
	Coefficient	Coefficient
$\sigma_{u}$ (between individual-variation)	9.9	10.3
$\sigma_{e}$ (residual: within-variation)	11.2	12.1
ρ [Intraclass correlation (ICC)]	0.4	0.4

**Table 3:** Variability of reported age at first sex (AFS) and marriage (AFS) among multiple reporters by age.

Variance components and Intraclass correlation (ICC)	AFS variability by age categories			
	15–19 years	20–24 years	15–24 years	25–49 years
	Responses = 2,999	Responses = 4,934	Responses = 12,783	Responses = 16,845
	Individuals = 2,550	Individuals = 4,004	Individuals = 10,519	Individuals = 8,033
	Coefficient			
$\sigma_{u}\;$(between individual-variation)	1.8	2.5	2.3	6.1
$\sigma_{e}$ (residual: within-variation)	2.0	3.1	2.7	8.3
ρ [Intraclass correlation (ICC)]	0.4	0.4	0.4	0.4
	AFM variability by age categories			
	Responses = 469	Responses = 2,205	Responses = 2,674	Responses = 13,268
	Individuals = 459	Individuals = 1,979	Individuals = 2,252	Individuals = 7,134
	Coefficient			
$\sigma_{u}$ (between individual-variation)	1.3	2.2	2.2	5.4
$\sigma_{e}$ (residual: within-variation)	2.4	1. 7	1.7	6.3
ρ [Intraclass correlation (ICC)]	0.2	0.6	0.6	0.4

### Consistency and inconsistencies in reported AFS and AFM among individuals with multiple reports

We examined the consistency and inconsistencies in reported AFS and AFM among individuals who reported eligible values more than once and ever experienced the events. We excluded those who reported these values only once or those who had never engaged in sexual activity or marriage.

 Table 4 provides an overview of the findings. Among the individuals who reported eligible AFS more than once and ever had sex (a total of 6,690 individuals), about 1,209 (18.1%) consistently provided the same values for AFS. For AFM, out of 4,527 individuals, approximately 954 (21.1%) reported consistent values. When we collapsed the levels, we included those who consistently reported values in most rounds except one or had commonly reported values in all rounds except a few. We grouped them with people who reported consistent values. In this context, we found that overall, 51.9% reported consistent AFS, and 56.1% reported consistent AFM (Table 4).

**Table 4:** Consistency in reported age at first sex (AFS) and marriage (AFS) among multiple reporters: (1994–2016).

Reported age at first sex (AFS) and age at first Marriage (AFM) consistencies in four level of categories: overall		
	AFS	AFM
	*N* = 6,690	*N* = 4,527
	*n* (%)	*n* (%)
Reported consistently	1,209 (18.1)	954 (21.1)
Inconsistent: can identify most likely age	1,069 (16.0)	922 (20.4)
Inconsistent: can be corrected	1,195 (17.9))	664 (14.7)
Inconsistent: cannot identify most likely age	3,217 (48.1)	1,987 (43.9)
Reported AFS and AFM consistencies in two level of categories (collapsed from four levels): Overall		
	AFS	AFM
	*N* = 6,690	*N* = 4,527
	*n* (%)	*n* (%)
Reports consistent	3,473 (51.9)	2,540 (56.1)
Reports inconsistent	3,217 (48.1)	1,987 (43.9)

Consistency by age groups showed that adolescents had higher consistency for both AFS and AFM compared to young adults (Table 5). Among the younger population (15–24 years), consistency was 49.8% for AFS and 67.5% for AFM. Reporting consistency for AFS and AFM varied slightly by education level, residence, and HIV status. However, females showed higher consistency (56.7%) than males (43.5%) for AFS and for female (61.0%) and males (44.9%) for AFM (Supplementary Table S3).

**Table 5:** Consistency in reported ages at first sex (AFS) and marriage (AFM) among multiple reporters by age: (1994–2016).

Reported consistency categories	Reported AFS consistency in four level of categories			
	Age categories**			
	15–19 years	20–24 years	25–49 years	15–24 years
	*N* = 1,177	*N* = 1,460	*N* = 3,475	*N* = 2,637
	*n* (%)	*n* (%)	*n* (%)	*n* (%)
Reported consistently	257 (21.8)	268 (18.4)	579 (16.7)	525 (19.9)
Inconsistent: can identify most likely age	115 (9.8)	191 (13.1)	656 (18.9)	306 (11.6)
Inconsistent: can be corrected	232 (19.7)	249 (17.1)	626 (18.0)	481 (18.2)
Inconsistent: cannot identify most likely age	573 (48.7)	752 (51.5)	1,614 (46.5)	1,325 (50.3)
Reported AFS consistency in two level of categories (collapsed from four levels)				
Reports consistent	604 (51.3)	708 (48.5)	1,861 (53.6)	1,312 (49.8)
Reports inconsistent	573 (48.7)	752 (51.5)	1,614 (46.5)	1,325 (50.3)
	Reported AFM consistency in four level of categories			
	Age categories**			
	15–19 years	20–24 years	25–49 years	15–24 years
	*N* = 188	*N* = 732	*N* = 2,947	*N* = 920
	*n* (%)	*n* (%)	*n* (%)	*n* (%)
Reported consistently	58 (30.9)	173 (23.6)	581 (19.7)	231 (25.1)
Inconsistent: can identify most likely age	28 (14.9)	151 (20.6)	631 (21.4)	179 (19.5)
Inconsistent: can be corrected	46 (24.5)	165 (22.5)	380 (12.9)	211 (22.9)
Inconsistent: cannot identify most likely age	56 (29.8)	243 (33.2)	1,355 (46.0)	299 (32.5)
Reported AFM consistency in two level of categories (collapsed from four levels)				
Reports consistent	132 (70.2)	489 (66.8)	1,592 (54.0)	621 (67.5)
Reports inconsistent	56 (29.8)	243 (33.2)	1,355 (46.0)	299 (32.5)

** Significant in both (in four and two levels) (*p*-value <0.05).

### Reliable and unreliable reported AFS and AFM data

Overall, out of 31,522 individuals who reported AFS data, 22,860 (72.5%) entries are considered reliable, while 8,662 (27.5%) are deemed unreliable even after correction of inconsistencies. Among the young population, 12,412 (80.9%) entries are classified as reliable data, while 2,938 (19.1%) are unreliable data. As for AFM data, 24,852 (78.6%) entries are marked as reliable data, with 6,775 (21.4%) labelled as unreliable. In the case of the young population, 14,245 (92.0%) AFM entries are reliable, and 1,241 (8.0%) are unreliable (Table 6).

**Table 6:** Age at first sex (AFS) and first marriage (AFM) reported data flags and usability (1994–2016).

All ages (15 years and above)			
Flag of AFS		Number of people with reliable data	Number of people with unreliable data
	*N* = 31,522	*N* = 22,860	*N* = 8,662
	*n* (%)		
Never had sex	4,941 (15.7)	✓	
Reported only in one round	14,446 (45.8)	✓	
All reports consistent	1,209 (3.8)	✓	
Reports differ by 1 year only	1,195 (3.8)	✓	
Reports differ by >1 year, used mean	3,217 (10.2)		✗
Used most frequently reported age	1,069 (3.4)	✓	
Already had sex on entry, no AFS reported	4,965 (15.8)		✗
Missing	480 (1.5)		✗
Flag of AFM		Number of people with reliable data	Number of people with unreliable data
	*N* = 31,627	*N* = 24,852	*N* = 6,775
	*n* (%)		
Never had marriage	11,553 (36.5)	✓	
Reported only in one round	10,759 (34.0)	✓	
All reports consistent	954 (3.0)	✓	
Reports differ by 1 year only	664 (2.1)	✓	
Reports differ by >1 year, used mean	1,987 (6.3)		✗
Used most frequently reported age	922 (2.9)	✓	
Already had marriage on entry, no AFM reported	4,505 (14.2)		✗
Missing	283 (0.9)		✗
15–24 years			
Flag of AFS		Number of people with reliable data	Number of people with unreliable data
	*N* = 15,350	*N* = 12,412	*N* = 2,938
	*n* (%)		
Never had sex	4,872 (31.7)	✓	
Reported only in one round	6,228 (440.6)	✓	
All reports consistent	525 (3.4)	✓	
Reports differ by 1 year only	481 (3.1)	✓	
Reports differ by >1 year, used mean	1,325 (8.6)		✗
Used most frequently reported age	306 (2.0)	✓	
Already had sex on entry, no AFS reported	1,493 (9.7)		✗
Missing	120 (0.8)		✗
		Number of people	Number of people
Flag of AFM		with reliable data	with unreliable data
	*N* = 15,486	*N* = 14,245	*N* = 1,241
	*n* (%)		
Never had marriage	10,759 (69.5)	✓	
Reported only in one round	2,865 (18.5)	✓	
All reports consistent	231 (1.5)	✓	
Reports differ by 1 year only	211 (1.4)	✓	
Reports differ by >1 year, used mean	299 (1.9)		✗
Used most frequently reported age	179 (1.2)	✓	
Already had marriage on entry, no AFM reported	906 (5.9)		✗
Missing	36 (0.2)		✗

✓ = reliable data; ✗ = unreliable data.

## Discussion

The study found substantial within-individual variability and inconsistencies in reported AFS and AFM when individuals were asked about these milestones on multiple occasions in Kisesa, North-West Tanzania. An intraclass correlation of 40% for AFS and AFM indicated that nearly half of the variation in reported AFS and AFM was due to changes individuals made over time, potentially influenced by changing circumstances. Overall, 51.9% of participants reported AFS consistently, and 56.1% reported AFM consistently, while 27.5% (8,662) of AFS data and 21.4% (6,775) of AFM data remained unreliable, even after correcting for inconsistencies. Among those aged 15–24 years, 49.8% consistently reported AFS, and 67.5% consistently reported AFM, with 19.1% (2,938) of AFS data and 8.0% (1,241) of AFM data deemed unreliable. Overall, in this analysis, AFM was consistently reported higher than AFS, especially among the younger population. However, it's important to consider that reporting consistency for AFM and AFS can still be influenced by various factors, including cultural norms and social desirability (44). While greater consistency in AFM reporting is a general trend in this analysis, it may not apply universally across all populations or cultural contexts. Previous studies have reported similar challenges. For instance, Wringe et al. (35) highlighted significant inconsistencies in age-at-event reporting across three HIV cohort studies in sub-Saharan Africa, while Nguyen (45) identified reporting biases in AFS data from 42 SSA countries, especially among adolescents.

The findings from the sub-analysis show that age, sex, residence area, HIV status, pregnancy status, and education level can impact the variability and consistency in reporting AFS and AFM, though only to a small degree. The most notable differences was for AFM, with females demonstrating higher consistency (61.0%) compared to males (44.9%). Minor differences were also observed across other variables, including residence area, HIV status, and pregnancy status. These results have important implications for the design and interpretation of studies relying on self-reported data, particularly in sexual and reproductive health. Researchers and policymakers should consider these demographic factors when assessing and addressing the reliability of such data.

Globally, the consistency of reported lifetime events varies (28, 31, 34, 35). A study conducted using two rounds of the National Longitudinal Study of Adolescent Health (Add Health) among those reporting sexual experience at both interviews found that only 22.2% reported the same age of first sex (31) and large variability in reporting ages. Reporting accuracy can be influenced by data collection methods; while audio computer-assisted self-interview (ACASI) has been shown to enhance privacy and accuracy (46), results are mixed. For example, in Kenya, ACASI increased reports of sensitive behaviours like coerced sex but did not significantly change reports of consensual sexual activity (30). In Malawi, where AFS was collected via ACASI and AFM via face-to-face interviews, only 36.6% of adolescents reported consistent ages between the two method (28). A study in India found that ACASI did not uniformly increase reporting levels compared to face-to-face interviews, with variations observed based on gender and the type of sexual behaviour reported (47). This shows that even with ACASI as a tool for collecting information, getting a higher proportion of consistent results in reporting the age of these events remains a challenge (30). Hence, a systematic analytical approach as the way implemented in this current study is essential for improving data quality and obtaining accurate estimates for better reporting.

Our study aligns with findings from other regions, where consistency levels were around 50.0% and above, both overall and in subgroups of the population (20, 26, 27, 31). A study conducted in Kenya reported a consistency level of around 50% (20), which was similar to the findings of this study. However, studies in higher-income settings, such as the Unites States and Australia, have reported higher consistency levels (above 80%) (26, 27, 31). Various factors could contribute to such differences between the study conducted in Africa and Europe, as we observed slight variations in the level of consistency based on demographics such as education level, sex, age, and residence area. These discrepancies could be influenced by traditional values and norms prevalent in developing countries like Kenya and Tanzania compared to developed countries like America. In Tanzania, previous research has shown sex- and cohort-specific variations in reporting consistency (48) and median AFS (49). Our findings extend this evidence, highlighting the variability and consistency of the reported AFS and AFM overall and specifically for adolescents and young adults. It also examines subgroup analysis results based on other demographics and HIV status. Additionally, the study identified data quality issues, suggesting that some data remain unreliable even after correcting for inconsistencies. These unreliable data likely resulted from inconsistent or unreliable responses. Qualitative research could be used to understand these variations and inconsistencies, helping to tailor interventions and policies for obtaining accurate information across different subgroups. One potential approach is conducting qualitative research with individuals to assess their ability to recall and describe their initial sexual experiences or marriages (24, 50). Rather than focusing on their exact age at the time, the emphasis should be on determining whether they remember when the event occurred, its significance in their memory, and their confidence in recalling it. Additionally, understanding the reasons behind gender-specific reporting inconsistencies is crucial for obtaining reliable data. This should involve exploring the sociocultural dynamics that influence data reporting among different gender groups (51).

## Strength and limitation of the study

The strength of the study lies in its comprehensive approach to observe variability and consistency of the reported AFS and AFM using longitudinal data which has eight rounds especially for adolescent and young adults. Through the utilization of a fixed-effects panel regression analysis, the study acknowledges the repeated responses nature of the data, enabling examination of both individual and between variability. Furthermore, the incorporation of sub-analysis analysis allows the study to identify variation of the results in different groups and their patterns which gives the information of how we can handle the quality of the reported ages target specific groups. This approach provides invaluable insights into the diverse needs and preferences of the target population. Additionally, this study provides valuable insights by presenting a systematic approach to assess these inconsistencies, offering guidance on data exclusion before progressing to the subsequent stages of analysis.

However, the study has some limitations. A small subset of individuals reported AFS and AFM across multiple rounds, which may not represent the broader population (Supplementary Tables S4, S5). Also, these surveys did not differentiate between formal marriage and cohabitation (living with a partner). This lack of distinction is significant as the legal status of marriage may influence individuals' behaviours, including sexual risk behaviours. For instance, individuals in formal marriages may exhibit fewer sexual risk behaviours compared to those cohabiting. The time gaps between the reports could also influence the results, as longer time gaps might increase recall errors. However, when restricting the analysis to intervals of two to three rounds (e.g., comparing the consistency of reported AFS in the first two survey rounds with the last three survey rounds), the differences were minimal, which did not change the overall conclusions. Additionally, while inconsistencies in AFS and AFM reporting are primarily attributed to individual recall and social desirability biases, we cannot rule out the potential influence of data collection and recording systems. Since this study relies on secondary data collected over multiple survey rounds from 1994 to 2016, verifying whether recording errors contributed to inconsistencies is not feasible without field validation. Finally, we did not estimate the median AFS and AFM to demonstrate the impact of our systematic analytical approach in correcting inconsistencies in the estimates. Future analyses will address this limitation by including the estimation of these indicators and comparing the results before and after addressing the inconsistencies.

## Conclusions

The findings highlight the challenge posed by self-reported AFS and AFM data due to inherent variability and inconsistency. A systematic approach is necessary to exclude or correct inconsistent data from the analysis and ensure respondents correctly understand the questions asked by interviewers. Public health programs or researchers may need to target specific populations and gather data from those groups to address variations and other challenges in collecting AFS and AFM data.

## Data Availability

The original contributions presented in the study are included in the article/Supplementary Material, further inquiries can be directed to the corresponding author.
