# Supplementary material for: Consistency in self-reported age at first sex and marriage among adolescents and young adults in Northwestern Tanzania: insights from repeated responses
Source: Front Reprod Health. 2025 Jun 12;7:1488604. doi: 10.3389/frph.2025.1488604 (PMC12198193; doi:10.3389/frph.2025.1488604)
Supplement: Supplementary file 1 [file Table1.docx]

**Supplementary Table 1:** Coding and explanation of created variables **^†^**

| **Variable** | **Description** | **Code value description for reported AFS and AFM** | **Action taken/words used** |
| --- | --- | --- | --- |
| Flag of AFS and AFM | Different scenarios reported by participants about AFS/AFM who attended at least once across survey rounds | 0= Never experienced the event (i.e., sex/marriage) and no AFS/AFM reported |  |
|  |  | 1= People who have only reported once | use that age across all rounds |
|  |  | 2=People who have reported more than once and were consistent (i.e., all reported values are | use the reported values |
|  |  | consistent) |  |
|  |  | 3= Reported values differ by 1 year only across the rounds | take the oldest reported age |
|  |  | 4= Reported values differ by more than 1 year | use the mean |
|  |  | 5= If only 1 report is different to the others across the rounds | use the common reported age, instead of mean |
|  |  |  | (i.e., use the youngest age if that was the mostly common |
|  |  |  | or change and use the oldest |
|  |  |  | age if that was the common reported ages) |
|  |  |  |  |
|  |  |  |  |
|  |  |  |  |
|  |  |  |  |
|  |  |  |  |
|  |  |  |  |
|  |  |  |  |
|  |  | 6=Respondents who had first sex before age 15 years in the date of interview and never reported AFS | Already had sex on entry, no AFS/AFM reported |
|  |  |  |  |
|  |  |  |  |
|  |  | 7= Respondents who haven't answered the questions about sex and | Missing |
|  |  | marriage |  |
|  |  |  |  |
| Reliable AFS/AFM data | Shows which AFS/AFM data are reliable for the future analysis | 1= Never experienced events (i.e., sex/marriage) and did not report their AFS or AFM multiple surveys, or reported AFS/AFM only once across eight surveys, or reported AFS/AFM multiple times but consistently, or only one survey value differs from the rest of the surveys (i.e., the most common one was considered as the valid reported value), or reported more than once and differed by only one year (i.e., the oldest age was considered) | 1= Reliable AFS/AFM data: These are reliable data that can be used for analysis after the correction of inconsistencies. |
|  |  |  |  |
|  |  | 0= Reported AFS/AFM multiple times but with variations exceeding one year (i.e., 2, 3, 4, 5, etc.) across the eight surveys (i.e., the mean value was considered as the common value), or the reported values require more observations by either going back to trace in the questionnaire or in the field to understand what actually happened due to high discrepancies (i.e., require edits by humans, which are not visible at this stage as the analysis uses secondary data which was already collected a few years ago), or the individuals reported already experiencing the events but the AFS/AFM was not presented | 0= Unreliable AFS/AFM data: These are data that are not reliable enough to be used for the analysis even after correction of the inconsistencies |
|  |  |  |  |
| Quality of reported AFS/AFM (stringent) | Shows the categories of the reported AFS/AFM qualities for only those reported more than once (i.e., stringent and details one) | 1=AFS/AFM reported more than once and are consistently | AFS/AFM: reported consistently |
|  |  | 2=AFS/AFM reported more than once and only one report is different from others | AFS/AFM: inconsistent: can identify most likely age |
|  |  | 3=AFS/AFM reported more than once and reports differ by 1 year only | AFS/AFM: inconsistent: can be corrected |
|  |  | 4=AFS/AFM reported more than once and reports differ by more than 1 year | AFS/AFM: cannot identify most likely age |
|  |  | 5=AFS/AFM reported more than once and never had sex or marriage/ reports needs edits by human/already had sex or marriage on entry | Others |
|  |  | no AFS/AFM reported |  |
|  |  |  |  |
| Quality of reported (relaxed) | Shows the categories of the reported AFS/AFM qualities for only those reported more than once (i.e., relaxed and collapsed in two levels) | 0= AFS/AFM reported more than once and reports differ by more than 1 year | Reported inconsistent |
|  |  | 1= AFS/AFM reported more than once and are consistently or AFS/AFM reported more than once and only one report is different from others or AFS/AFM reported more than once and reports differ by 1 year only | Reported consistent |

**^†^**The variables in this table represent separate indicators for AFS and AFM during the analysis.
