# Supplementary material for: Consistency in self-reported age at first sex and marriage among adolescents and young adults in Northwestern Tanzania: insights from repeated responses
Source: Front Reprod Health. 2025 Jun 12;7:1488604. doi: 10.3389/frph.2025.1488604 (PMC12198193; doi:10.3389/frph.2025.1488604)
Supplement: Supplementary file 2 [file Table2.docx]

**Supplementary Table 2:** Variability of reported age at first sex (AFS) and marriage (AFS) among multiple reporters by demographics and HIV Status

|  | **AFS variability by demographics and HIV Status** | | | | | | |  |  |  |
| --- | --- | --- | --- | --- | --- | --- | --- | --- | --- | --- |
|  | **Sex** | | | |  | **Education level** | |  | **Residence area** | |
|  | **Male** | | **Female** | | **No** | **Primary** | | **Secondary or** | **Rural** | **Semi-urban** |
|  |  | |  | | **education** | **education** | | **higher education** |  |  |
|  | Responses=11,997 | | Responses=17,994 | | Responses= 8,850 | Responses=18,818 | | Responses=2,268 | Responses=19,069 | Responses=10,920 |
|  | Individuals=5,419 | | Individuals=7,478 | | Individuals=4,461 | Individuals=9,011 | | Individuals=1,479 | Individuals=8,472 | Individuals=5,184 |
|  | **Coefficient** | |  | |  |  | |  |  |  |
| $\sigma_{u}$(between individual-variation) | 9.83 | | 9.86 | | 14.61 | 7.11 | | 7.52 | 10.69 | 9.11 |
| $\sigma_{e}$(residual: within-variation) | 11.76 | | 10.81 | | 16.11 | 8.52 | | 7.51 | 11.32 | 10.61 |
| $\rho$(Intraclass correlation (ICC)) | 0.41 | | 0.45 | | 0.45 | 0.41 | | 0.50 | 0.47 | 0.42 |
|  | **AFM variability by demographics and HIV Status** | | | | | | |  |  |  |
|  | **Sex** | |  | |  | **Education level** | | | **Residence area** |  |
|  | **Male** | | **Female** | | **No** | **Primary** | | **Secondary or** | **Rural** | **Semi-urban** |
|  |  | |  | | **education** | **education** | | **higher education** |  |  |
|  | Responses=6,954 | | Responses=14,559 | | Responses=7,679 | Responses=12,794 | | Responses988 | Responses=13,699 | Responses=7,813 |
|  | Individuals=3,473 | | Individuals=6,639 | | Individuals=3,956 | Individuals=6,782 | | Individuals=645 | Individuals=6,649 | Individuals=3,989 |
|  | Coefficient | |  | |  |  | |  |  |  |
| $\sigma_{u}$(between individual-variation) | 8.31 | | 10.92 | | 14.96 | 6.47 | | 6.81 | 10.60 | 10.46 |
| $\sigma_{e}$(residual: within-variation) | 11.36 | | 12.47 | | 18.17 | 6.98 | | 6.27 | 12.52 | 10.99 |
| $\rho$(Intraclass correlation (ICC)) | 0.35 | | 0.43 | | 0.40 | 0.46 | | 0.54 | 0.42 | 0.47 |
| **Continuation: for AFS** |  |  | |  | | |  |  |  |  |
|  | **Pregnancy** | | | **HIV status** | | | |  |  |  |
|  | **Never** | **Ever** | | **Negative** | | | **Positive** |  |  |  |
|  |  |  | |  | | |  |  |  |  |
|  | Responses= 1,405 | Responses=15,613 | | Responses=23,921 | | | Responses=1,615 |  |  |  |
|  | Individuals=1,111 | Individuals=6,877 | | Individuals=11,997 | | | Individuals=1,098 |  |  |  |
|  |  |  | |  | | |  |  |  |  |
| $\sigma_{u}$(between individual-variation) | 6.99 | 9.13 | | 10.23 | | | 9.11 |  |  |  |
| $\sigma_{e}$(residual: within-variation) | 6.08 | 9.99 | | 11.53 | | | 10.56 |  |  |  |
| $\rho$(Intraclass correlation (ICC)) | 0.57 | 0.45 | | 0.44 | | | 0.43 |  |  |  |
|  |  |  | |  | | |  |  |  |  |
|  |  |  | |  | | |  |  |  |  |
| **Continuation: for AFM** | **Pregnancy** | | | **HIV status** | | | |  |  |  |
|  | **Never** | **Ever** | | **Negative** | | | **Positive** |  |  |  |
|  |  |  | |  | | |  |  |  |  |
|  | Responses=352 | Responses=13,088 | | Responses=16,331 | | | Responses=1,206 |  |  |  |
|  | Individuals=296 | Individuals=6,355 | | Individuals=9,133 | | | Individuals=891 |  |  |  |
|  |  |  | |  | | |  |  |  |  |
| $\sigma_{u}$(between individual-variation) | 10.31 | 10.17 | | 10.54 | | | 8.75 |  |  |  |
| $\sigma_{e}$(residual: within-variation) | 12.01 | 10.91 | | 12.54 | | | 10.32 |  |  |  |
| $\rho$(Intraclass correlation (ICC)) | 0.42 | 0.46 | | 0.41 | | | 0.42 |  |  |  |
