# Supplementary material for: Consistency in self-reported age at first sex and marriage among adolescents and young adults in Northwestern Tanzania: insights from repeated responses
Source: Front Reprod Health. 2025 Jun 12;7:1488604. doi: 10.3389/frph.2025.1488604 (PMC12198193; doi:10.3389/frph.2025.1488604)
Supplement: Supplementary file 3 [file Table3.docx]

**Supplementary Table 3 Consistency in reported ages at first sex (AFS) and marriage (AFM) among multiple reporters: by demographics characteristics and HIV Status (1994-2016)**

|  | | **Reported age at first sex (AFS) consistency in four level of categories** | | | | | | | | | | | | | | | | | | | | |  |
| --- | --- | --- | --- | --- | --- | --- | --- | --- | --- | --- | --- | --- | --- | --- | --- | --- | --- | --- | --- | --- | --- | --- | --- |
|  | | **Sex^**^** | |  | |  | | **Education level^*^** | | | **Residence area^*^** | | | | **Pregnancy^**^** | | | | **HIV status^†^** | | | |  |
|  | | Male | | Female | | No | | Primary | Secondary or | | Rural | | Semi-urban | | Never | | Ever | | Negative | | Positive | |  |
|  | |  | |  | | education | | education | higher education | |  | |  | |  | |  | |  | |  | |  |
|  | | **N=2,427** | | **N=4,263** | | **N=1,671** | | **N=4,667** | **N=350** | | **N=3,979** | | **N=2,711** | | **N=524** | | **N=3,664** | | **N=5770** | | **N=303** | |  |
|  | | n (%) | | n (%) | | n (%) | | n (%) | n (%) | | n (%) | | n (%) | | n (%) | | n (%) | | n (%) | | n (%) | |  |
| Reported consistently | | 376 (15.5) | | 833 (19.5) | | 324 (19.4) | | 806 (17.3) | 79 (22.6) | | 700 (17.6) | | 509 (18.8) | | 114 (21.8) | | 700 (19.1) | | 1015 (17.6) | | 52 (17.2) | |  |
| Inconsistent: can identify most likely age | | 302 (12.4) | | 767 (18.0) | | 266 (15.9) | | 767 (16.4) | 36 (10.3) | | 668 (16.8) | | 401 (14.8) | | 58 (11.1) | | 698 (19.1) | | 958 (16.6) | | 34 (11.2) | |  |
| Inconsistent: can be corrected | | 377 (15.5) | | 818 (19.2) | | 301 (18.0) | | 835 (17.9) | 58 (16.6) | | 673 (16.9) | | 522 (19.3) | | 96 (18.3) | | 708 (19.3) | | 996 (17.3) | | 61 (20.1) | |  |
| Inconsistent: cannot identify most likely age | | 1372 (56.5) | | 1845 (43.3) | | 780 (46.7) | | 2259 (48.4) | 177 (50.6) | | 1938 (48.7) | | 1279 (47.2) | | 256 (48.9) | | 1558 (42.5) | | 2801 (48.5) | | 156 (51.5) | |  |
|  | | **Reported AFS consistency in two level of categories (collapsed from four levels)** | | | | | | | | | | | | | | | | | | | | |  |
|  | |  | |  | |  | |  |  | |  | |  | |  | |  | |  | |  | |  |
| Reports consistent | | 1055 (43.5) | | 2418 (56.7) | | 891 (53.3) | | 2408 (51.6) | 173 (49.4) | | 2041 (51.3) | | 1432 (52.8) | | 268 (51.2) | | 2106 (57.5) | | 2969 (51.5) | | 147 (48.5) | |  |
| Reports inconsistent | | 1372 (56.5) | | 1845 (43.3) | | 780 (46.7) | | 2259 (48.4) | 177 (50.6) | | 1938 (48.7) | | 1279 (47.2) | | 256 (48.9) | | 1558 (42.5) | | 2801 (48.5) | | 156 (51.5) | |  |
|  | **Reported age at first marriage (AFM) consistency in four level of categories** | | | | | | | | | | | | | | | | | | | | | | |
|  | **Sex^**^** | |  | |  | | **Education level^**^** | | | | | **Residence area^*^** | | | | **Pregnancy^†^** | | | | **HIV status^*^** | | | |
|  | Male | | Female | | No | | Primary | | | Secondary or | | Rural | | Semi-urban | | Never | | Ever | | Negative | | Positive | |
|  |  | |  | | education | | education | | | higher education | |  | |  | |  | |  | |  | |  | |
|  | **N=1,377** | | **3,150** | | **N=1,333** | | **N=3,017** | | | **N=174** | | **N=2,672** | | **N=1,855** | | **N=105** | | **N=2,963** | | **N=3,789** | | **N=195** | |
|  | n (%) | | n (%) | | n (%) | | n (%) | | | n (%) | | n (%) | | n (%) | | n (%) | | n (%) | | n (%) | | n (%) | |
| Reported consistently | 207 (15.0) | | 747 (23.7) | | 249 (18.7) | | 659 (21.8) | | | 46 (26.4) | | 527 (19.7) | | 427 (23.0) | | 26 (24.8) | | 697 (23.5) | | 746 (19.7) | | 48 (24.6) | |
| Inconsistent: can identify most likely age | 251 (18.2) | | 671 (21.3) | | 229 (17.2) | | 671 (22.2) | | | 21 (12.1) | | 569 (21.3) | | 353 (19.0) | | 22 (21.0) | | 634 (21.4) | | 826 (21.8) | | 25 (12.8) | |
| Inconsistent: can be corrected | 160 (11.6) | | 504 (16.0) | | 207 (15.5) | | 424 (14.1) | | | 32 (18.4) | | 379 (14.2) | | 285 (15.4) | | 18 (17.1) | | 474 (16.0) | | 539 (14.2) | | 25 (12.8) | |
| Inconsistent: cannot identify most likely age | 759 (55.1) | | 1228 (39.0) | | 648 (48.6) | | 1263 (41.9) | | | 75 (43.1) | | 1197 (44.8) | | 790 (42.6) | | 39 (37.1) | | 1158 (39.1) | | 1678 (44.3) | | 97 (49.7) | |
|  | **Reported AFM consistency in two level of categories (collapsed from four levels)** | | | | | | | | | | | | | | | | | | | | | | |
|  |  | |  | |  | |  | | |  | |  | |  | |  | |  | |  | |  | |
| Reports consistent | 618 (44.9) | | 1922 (61.0) | | 685 (51.4) | | 1754 (58.1) | | | 99 (56.9) | | 1475 (55.2) | | 1065 (57.4) | | 66 (62.9) | | 1805 (60.9) | | 2111 (55.7) | | 98 (50.3) | |
| Reports inconsistent | 759 (55.1) | | 1228 (39.0) | | 648 (48.6) | | 1263 (41.9) | | | 75 (43.1) | | 1197 (44.8) | | 790 (42.6) | | 39 (37.1) | | 1158 (39.1) | | 1678 (44.3) | | 97 (49.7) | |

^**^Significant in both (in four and two levels) (p-value<0.05); ^*^Significant in four levels only (p-value <0.05); **^†^**Insignificant in both (in four and two levels) (p-value>0.05)
