# Supplementary material for: Consistency in self-reported age at first sex and marriage among adolescents and young adults in Northwestern Tanzania: insights from repeated responses
Source: Front Reprod Health. 2025 Jun 12;7:1488604. doi: 10.3389/frph.2025.1488604 (PMC12198193; doi:10.3389/frph.2025.1488604)
Supplement: Supplementary file 4 [file Table4.docx]

**Supplementary Table 4: Survey responses, age at first sex (AFS), and first marriage (AFM) (1994-2016)**

| **Survey rounds** | | No. of responses | | No. of responses | | No. of responses | | No. of responses | | No. of responses | | No. of responses | | No. of responses | |
| --- | --- | --- | --- | --- | --- | --- | --- | --- | --- | --- | --- | --- | --- | --- | --- |
|  | |  | | ever had sex | | never had sex | | AFS | | ever married | | never been married | | AFM | |
| 1 [1994/1995] | | 5785 | | 5027 | | 758 | | 4764 | | 3701 | | 2084 | | 3484 | |
| 2 [1996/1997] | | 6362 | | 5812 | | 535 | | 4029 | | 4336 | | 2026 | | 0 | |
| 3 [1999/2000] | | 7338 | | 6697 | | 641 | | 0 | | 5480 | | 1858 | | 0 | |
| 4 [2003/2004] | | 8895 | | 7899 | | 658 | | 7544 | | 6154 | | 2380 | | 5525 | |
| 5 [2006/2007] | | 8272 | | 7126 | | 1133 | | 5594 | | 5680 | | 2579 | | 4686 | |
| 6 [2010] | | 7668 | | 6192 | | 1418 | | 4934 | | 5114 | | 2524 | | 4113 | |
| 7 [2012/2013] | | 7228 | | 5877 | | 1351 | | 4448 | | 4835 | | 2391 | | 3899 | |
| 8 [2015/2016] | | 7106 | | 5795 | | 1311 | | 4467 | | 4772 | | 2334 | | 3920 | |
| **Total (N)** | | **58,654** | | **50,425** | | **7,805** | | **35,779** | | **40,072** | | **18,176** | | **25,627** | |
| **Individual (N)** | | **33,177** | | **28,354** | | **6,873** | | **23,489** | | **22,090** | | **13,451** | | **17,068** | |
| **Missing: Total** | |  | | **n=424** | | | | **n=14,646** | | **n=406** | | | | **n=14,445** | |
| **Missing: Individual** | |  | | **n=147** | | | | **n=4,865** | | **n=141** | | | | **n=5,022** | |
|  | |  | | **15-24 years** | | | |  | |  | | | |  | |
| **Survey rounds** | | No. of responses | | No. of responses | | No. of responses | | No. of responses | | No. of responses | | No. of responses | | No. of responses | |
|  | |  | | ever had sex | | never had sex | | AFS | | ever married | | never been married | | AFM | |
| 1 [1994/1995] | | 2674 | | 1928 | | 746 | | 1803 | | 825 | | 1849 | | 786 | |
| 2 [1996/1997] | | 2799 | | 2267 | | 521 | | 2149 | | 1009 | | 1790 | | 0 | |
| 3 [1999/2000] | | 2582 | | 1952 | | 630 | | 0 | | 949 | | 1633 | | 0 | |
| 4 [2003/2004] | | 3244 | | 2590 | | 643 | | 2430 | | 1097 | | 2102 | | 1068 | |
| 5 [2006/2007] | | 3248 | | 2127 | | 1118 | | 2009 | | 873 | | 2368 | | 844 | |
| 6 [2010] | | 3190 | | 1777 | | 1366 | | 1663 | | 811 | | 2360 | | 761 | |
| 7 [2012/2013] | | 2761 | | 1505 | | 1256 | | 1391 | | 666 | | 2094 | | 639 | |
| 8 [2015/2016] | | 2701 | | 1405 | | 1296 | | 1339 | | 620 | | 2081 | | 609 | |
| **Total (N)** | | **23,199** | | **15,551** | | **7,578** | | **12,783** | | **6,850** | | **12,340** | | **4707** | |
| **Individual (N)** | | **16,899** | | **12,068** | | **6,678** | | **10,519** | | **5,811** | | **11,049** | | **4,320** | |
| **Missing: Total** | |  | | **n=70** | | | | **n=2,771** | | **n=74** | | | | **n=2,143** | |
| **Missing: Individual** | |  | | **n=37** | | | | **n=1,549** | | **n=39** | | | | **n=1,491** | |
