# Supplementary material for: Consistency in self-reported age at first sex and marriage among adolescents and young adults in Northwestern Tanzania: insights from repeated responses
Source: Front Reprod Health. 2025 Jun 12;7:1488604. doi: 10.3389/frph.2025.1488604 (PMC12198193; doi:10.3389/frph.2025.1488604)
Supplement: Supplementary file 5 [file Table5.docx]

**Supplementary Table 5: Number of times people reported age at first sex (AFS) and first marriage (AFM) 1994-2016**

|  | **All ages (15+years)** | | **Aged 15-24 years** | |
| --- | --- | --- | --- | --- |
| **Number of reports** | **AFS** | **AFM** | **AFS** | **AFM** |
|  | **Responses (N=35,779)** | **Responses (N=25,627)** | **Responses (N=12,783)** | **Responses (N=4707)** |
|  | n (%) | n (%) | n (%) | n (%) |
| 1 | 15637 (43.7) | 11850 (46.2) | 8393 (65.7) | 3955 (84.0) |
| 2 | 10124 (28.3) | 6174 (24.1) | 3986 (31.2) | 686 (14.6) |
| 3 | 5082 (14.2) | 3801 (14.8) | 384 (3.0) | 66 (1.40) |
| 4 | 2836 (7.9) | 2284 (8.9) | 20 (0.2) |  |
| 5 | 1285 (3.6) | 1200 (4.7) |  |  |
| 6 | 570 (1.6) | 318 (1.2) |  |  |
| 7 | 245 (0.7) |  |  |  |
